# Precise detection of *Eimeria* oocysts in sheep: a deep learning model based on microscopic images

**DOI:** 10.1186/s13071-025-07092-4

**Published:** 2025-11-12

**Authors:** Liangliang Liu, Jinpu Xie, Huikai Qin, Xiangqing Sui, Longxian Zhang

**Affiliations:** 1 College of Veterinary Medicine, Henan Agricultural University, No. 15 Longzihu University Area, Zhengdong New District, Zhengzhou, Henan Province 450046 People’s Republic of China; 2 International Joint Research Laboratory for Zoonotic Diseases of Henan, Zhengzhou, 450046 Henan Province People’s Republic of China; 3 Key Laboratory of Quality and Safety Control of Poultry Products (Zhengzhou), Ministry of Agriculture and Rural Affairs, Beijing, People’s Republic of China

**Keywords:** *Eimeria* infection, Parasite oocysts, Deep learning, Object detection, YOLOv5

## Abstract

**Background:**

Parasitic infections remain a major cause of productivity loss in global livestock production. Traditional microscopic diagnostic methods are labor-intensive and require specialized veterinary expertise. Recent automated detection systems are hindered by limited annotated microscopy datasets and the difficulty of extracting discriminative features from small, overlapping targets.

**Methods:**

We propose YOLO-GA, an enhanced object detection framework, for accurate identification of *Eimeria* oocysts in ovine microscopy images. Built upon the YOLOv5’s architecture, the model incorporates two lightweight attention modules: (1) Contextual Transformer (CoT) blocks for local–global contextual enhancement and (2) Normalized Attention Mechanisms (NAM) for adaptive feature recalibration. The proposed model is optimized for both accuracy and computational efficiency.

**Results:**

Experiments on a curated dataset of 2000 microscopy images (200× magnification) demonstrated that YOLO-GA achieves a mean (± standard deviation) average precision (mAP@0.5) of 98.9% ± 0.1, with 95.2% ± 0.3 precision and real-time inference speed. Comparative evaluations against recent detectors, including YOLOv8, YOLOv10 and DETR variants, confirmed the superior performance of YOLO-GA across multiple runs.

**Conclusions:**

YOLO-GA offers a high-accuracy solution with balanced computational efficiency for automated detection of *Eimeria* oocysts under complex microscopy conditions. This work lays a foundation for intelligent diagnostics of ovine* Eimeria* coccidiosis and provides a reference for scalable health monitoring of sheep flocks, with potential extension to other small ruminant coccidiosis (e.g. goat* Eimeria*) pending further validation.

**Graphical Abstract:**

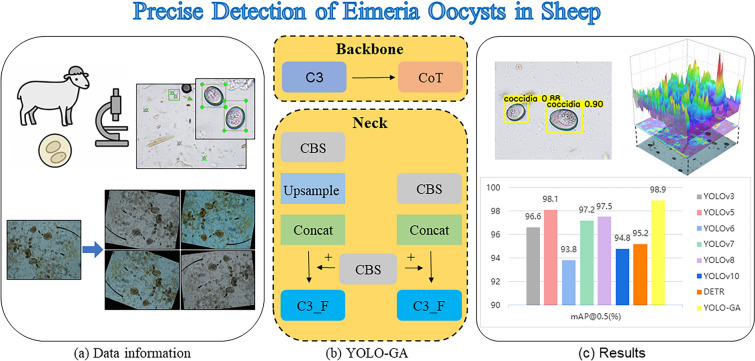

**Supplementary Information:**

The online version contains supplementary material available at 10.1186/s13071-025-07092-4.

## Background

*Eimeria* coccidiosis, caused by *Eimeria* spp. protozoa colonizing ovine intestinal epithelium, is a globally significant parasitic disease impairing lamb health and growth trajectories [1]. Transmission occurs predominantly via fecal–oral contamination cycles, wherein infected hosts shed sporulated oocysts into the environment, thereby establishing persistent epidemiological reservoirs [2] and resulting in regional, seasonal and clustered outbreaks. Epidemiological surveillance data reveal prevalence rates of 43.2–93.8% in Chinese sheep populations and 35.8–91.5% in goats (*n* = 12,347) [3]. Globally, similar infection patterns have been reported in countries such as Brazil [4], India [5] and Colombia [6]. Coccidiosis induces gastrointestinal inflammation, disrupts intestinal integrity and leads to malnutrition, causing weight loss, anemia, stunted growth, reduced feed efficiency and even mortality in severe cases [7]. These effects diminish economic returns and hinder the sustainable development of sheep farming [8]. Consequently, accurate detection of coccidia oocysts is critical for disease management.

Current diagnostic methods rely on microscopic examination by experienced experts to identify parasite oocysts, eggs or adult forms in samples [9]. However, this approach is labor-intensive, time-consuming and subjective. Recent advancements in artificial intelligence (AI) have revolutionized image analysis, offering novel solutions for disease diagnosis in both human and veterinary medicine [10]. For example, Marletta et al. [11] demonstrated AI-assisted rapid detection of malaria, bacteria and protozoa, and Kang et al. [12] integrated hyperspectral microscopy with bidirectional long short-term memory (LSTM) networks, attaining 92.9% classification accuracy (95% confidence interval [CI] 91.2–94.1%) for Salmonella-contaminated samples, outperforming traditional principal component analysis (PCA) classifiers. Zhang et al. [13] highlighted AI’s potential in enhancing pathogen identification accuracy, drug discovery and personalized treatment, despite challenges in data quality and model interpretability.

Building on these advancements, rapid and precise detection of parasites in microscopic images has become pivotal for livestock disease prevention. Deep learning, with its automated feature extraction, end-to-end optimization and high detection accuracy, has transformed object detection into two main paradigms: two-stage methods (e.g. R-CNN, Fast R-CNN, Faster R-CNN) and single-stage methods (e.g., YOLO series, SSD, RetinaNet).

Two-stage architectures employ region proposal networks (RPNs) for candidate region generation, followed by cascaded classification–regression modules. A representative example is Faster R-CNN, which generates candidate regions through a RPN and subsequently refines their classifications and bounding boxes. Although two-stage object detectors such as Faster R-CNN have been widely used in parasite detection, their computational overhead limits their applicability in real-time scenarios. Therefore, this study focuses on the development and comparison of single-stage detectors.

Single-stage detectors, typified by the YOLO series, directly predict object categories and bounding boxes in a single forward pass, offering superior efficiency and real-time capabilities. By framing detection as an end-to-end regression task, YOLO models exhibit robustness against complex backgrounds and are well-suited for resource-constrained environments. Recent advancements underscore their utility in parasitology. Koirala et al. [14] developed YOLO-mp, a three-layer streamlined architecture (21.8 BFLOPs) that achieves 94.07% mean average precision (mAP@0.5), outperforming standard YOLOv4 by 1.51% while reducing memory consumption by 37%. Mura et al. [15] introduced Ghost convolutions and a P2 prediction head in YOLO-Tryppa, compressing computational graphs (42% floating-point operations [FLOPs] reduction) and elevating trypanosome detection to 71.3% AP50. Rocha et al. [16] enhanced malaria detection to 96.32% mAP through refined multi-scale feature fusion strategies. Notably, Zedda et al. [17] pioneered the integration of transformers for global dependency modeling, attaining 83.6% mAP on the MP-IDB dataset, while Li et al. [18] utilized transfer learning to achieve 95.41% mAP for fish parasite detection with 80% frozen parameters. Large-scale validations further highlight their efficacy: Huo et al. [19] reported 99.4% accuracy in fecal sample analysis (*n* = 15,600), and Naing et al. [20] met WHO sensitivity benchmarks (95.08%) for protozoan screening. However, single-stage methods may exhibit slightly lower precision in densely cluttered or occluded scenarios compared to their two-stage counterparts.

To address these challenges, this study proposes YOLO-GA (YOLO with Globally-aware Attention), a high-speed, high-accuracy model for detecting *Eimeria* oocysts in microscopic images. YOLO-GA integrates YOLO’s efficiency, transformers for global dependency modeling and a normalization-based attention mechanism (NAM) for small object detection. Key contributions include:

1. We propose a novel globally-aware attention-enhanced detection model, YOLO-GA, for the precise identification of *Eimeria* oocysts in microscopic images. Unlike conventional YOLO-based frameworks, our model integrates Contextual Transformer (CoT) modules into the backbone for global context modeling and Normalized Attention Modules (NAM) into the neck to enhance small object detection under complex backgrounds.
2. We develop a feature refinement strategy that combines static convolutional features with dynamic attention mechanisms to improve small target localization and recognition accuracy. The CoT module strengthens the model’s ability to capture both local details and global semantic dependencies, while the NAM module adaptively recalibrates attention across channels and spatial dimensions, significantly improving precision and recall.
3. We validate the model’s effectiveness through comprehensive experiments and visualization analyses, including expert-annotated region comparisons and three-dimensional (3D) class activation mapping. These analyses demonstrate a high degree of consistency between the model’s attention regions and the diagnostic focus areas of veterinary experts, supporting the model’s reliability for clinical applications.

The remainder of this paper is organized as follows: The Methods section introduces the dataset, the improved YOLO-GA model architecture and the corresponding experimental setup. The Results and discussion section presents the training outcomes of the coccidian oocyst detection task, compares the proposed model with other state-of-the-art approaches, and employs heatmap-based visual analysis to explore the consistency between the model’s attention regions and the areas annotated by veterinary experts.

## Methods

### Dataset construction

**Fig. 1:**
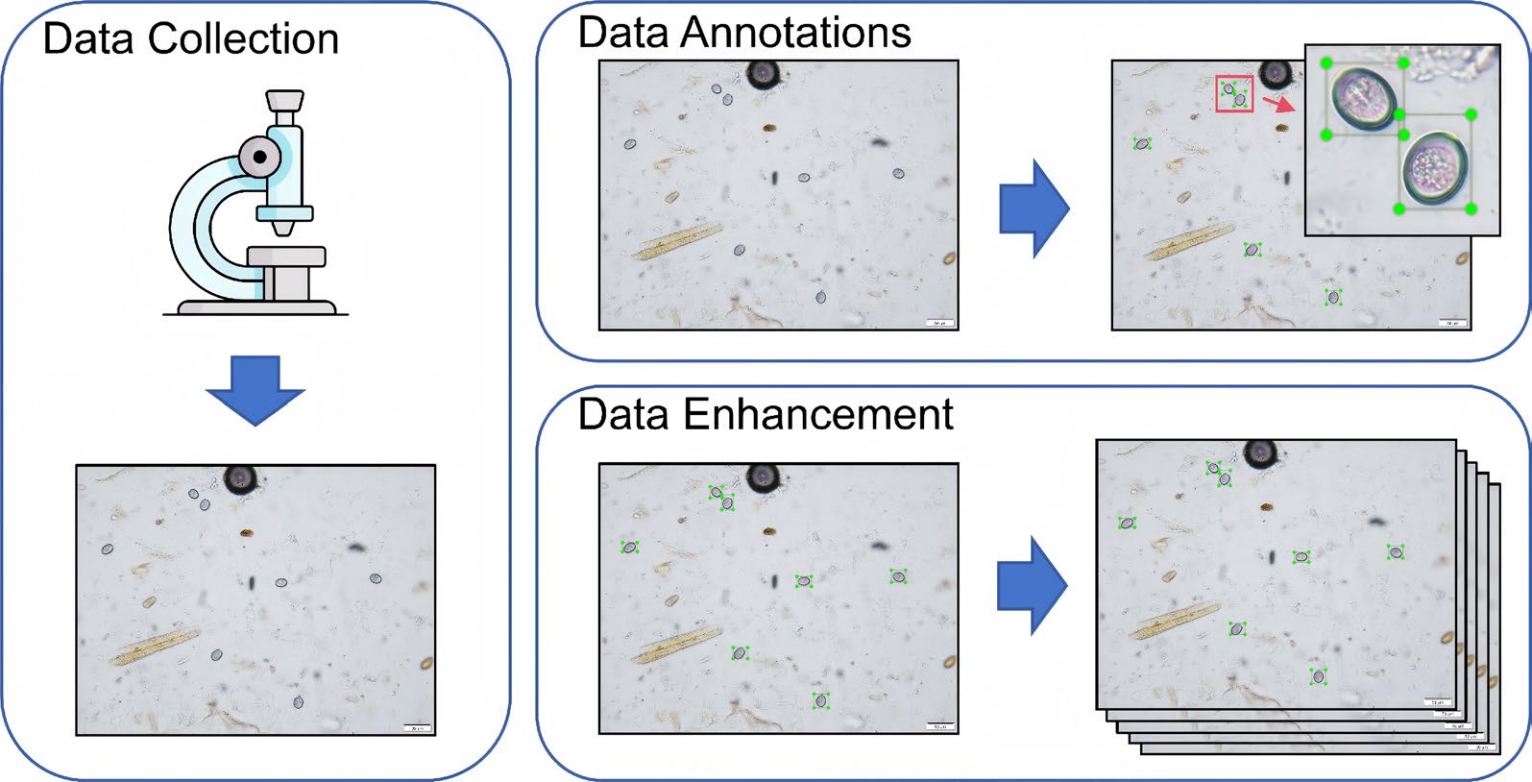
Workflow of dataset construction

The construction of the *Eimeria* oocyst image dataset involved three key stages: data collection, annotation and augmentation, as illustrated in Fig. 1.

First, a large number of fecal samples were collected from a sheep farm. Microscopic images of these samples were captured using a digital microscope at 200× magnification by veterinary researchers. A total of 500 images were acquired, representing *Eimeria* oocysts under varying visual conditions, including differences in lighting, background complexity and oocyst morphology. Although the samples were collected from sheep with varying levels of infection severity, infection severity refers to the distribution density and visual diversity of the oocysts rather than differences in parasite species or detection classes.

**Fig. 2:**
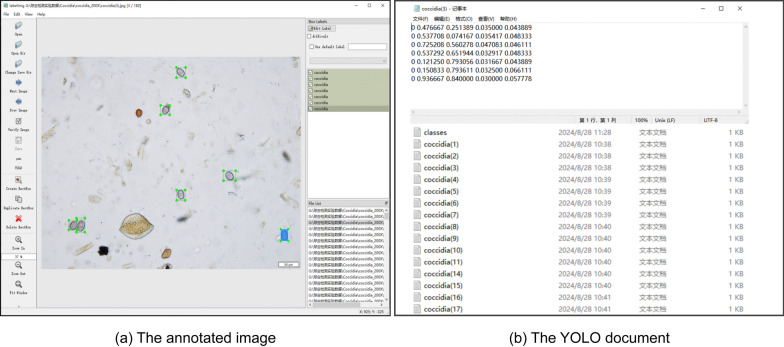
Example of *Eimeria* oocyst annotation under a microscope

Annotation was a critical step in the data preprocessing pipeline, as it directly influences both the training data quality and the model’s performance. To ensure accuracy and consistency, all images were manually labeled using the LabelImg tool by two independent veterinary researchers. Additionally, 20% of the labeled images were randomly selected to assess inter-annotator consistency, following standard practices in medical imaging datasets to balance verification reliability and annotation efficiency [21]. As shown in Fig. 2, Each oocyst in the images was carefully enclosed with a bounding box and uniformly labeled as “*Eimeria* oocyst,” which is the single class in this study. The boxes were tightly drawn around the oocyst boundaries to help the model accurately learn both positional and morphological features. All annotations were reviewed by experienced biomedical experts and trained annotators to ensure high-quality labeling. The annotation files were saved in YOLO format, with each file containing information about multiple bounding boxes per image. Each bounding box included the class index and normalized coordinates: the* x* and* y* coordinates of the box center, as well as its width and height. This standardized format facilitates efficient integration into YOLO-based detection models.

To improve data diversity and enhance the model’s generalization ability, we applied various data augmentation techniques exclusively to the training set. The validation and test sets remained unaltered to prevent data leakage and ensure fair evaluation. These included random rotations, scaling (from 0.8× to 1.2×), horizontal and vertical flipping, brightness and contrast adjustments, the addition of random noise and slight perturbations in color saturation. These augmentations simulated the variability in lighting conditions, imaging angles and microscope settings, helping to reduce overfitting and improve the model’s robustness in real-world scenarios. Representative examples of original and augmented images are shown in Additional file 1: Figure S1.

In total, the finalized dataset consisted of 2000 microscopic images containing 4215 annotated *Eimeria* oocysts, with an average of approximately 2.1 oocysts per image. This target density is appropriate for training small-object detection models. Representative samples are shown in Additional file 2: Figure S2. This high-quality, well-annotated dataset provides a solid foundation for training and evaluating the proposed detection model.

### Label distribution and correlation analysis

In addition to dataset annotation, we also performed a statistical analysis of label distribution and correlations to better understand the data characteristics. The results are shown in Additional file 3: Figure S3 and Additional file 4: Figure 4.

Following model training, we analyzed the annotated bounding box information and associated statistical features for the *Eimeria* oocysts in the dataset. These include class labels, bounding box size distribution, normalized coordinates of object centers, aspect ratio distributions and pairwise correlations among various features. Additional file 3: Figure S3a presents the data volume and number of categories in the training set, while Additional file 3: Figure S3b displays the size and count of annotated bounding boxes. Additional file 3: Figures S3c and S3d show the normalized distribution of bounding box center points and the height–width ratio distribution of target objects, respectively. In Additional file 3: Figures S3a and S3c, it is evident that the dataset contains a single class with over 400 instances. This sufficiently large sample size allows the model to better learn and fit the features of this category. Additionally, the spatial distribution of targets is relatively uniform and random across the image, with no apparent positional bias. It can also be observed in Additional file 3: Figures S3b and S3d that the bounding boxes are generally small and consistent in size. The width and height of the objects are positively correlated, indicating stable aspect ratios and homogeneous shapes. This distribution is advantageous for small-object detection tasks, as the model can focus on learning consistent shape and scale features.

In terms of correlation analysis, Additional file 4: Figure S4 visualizes the relationships among the center coordinates (*x*, *y*) of the bounding boxes and their dimensions (width, height). The first row shows the distribution of the *x*-coordinate of the object centers. The second row presents the relationship between *x* and *y*, as well as the distribution of *y*-coordinates. The third row illustrates how bounding box width correlates with *x* and *y*, along with its distribution. The fourth row shows how height correlates with *x*, *y* and width, as well as its own distribution. In Additional file 4: Figure S4, we observe that object location (*x*, *y*) shows no significant correlation with object size (width, height), indicating that the spatial position of a target does not influence its size. However, the strong positive correlation between width and height provides a reliable basis for the model to learn stable shape features of the target.

### Overview of model architecture

**Fig. 3:**
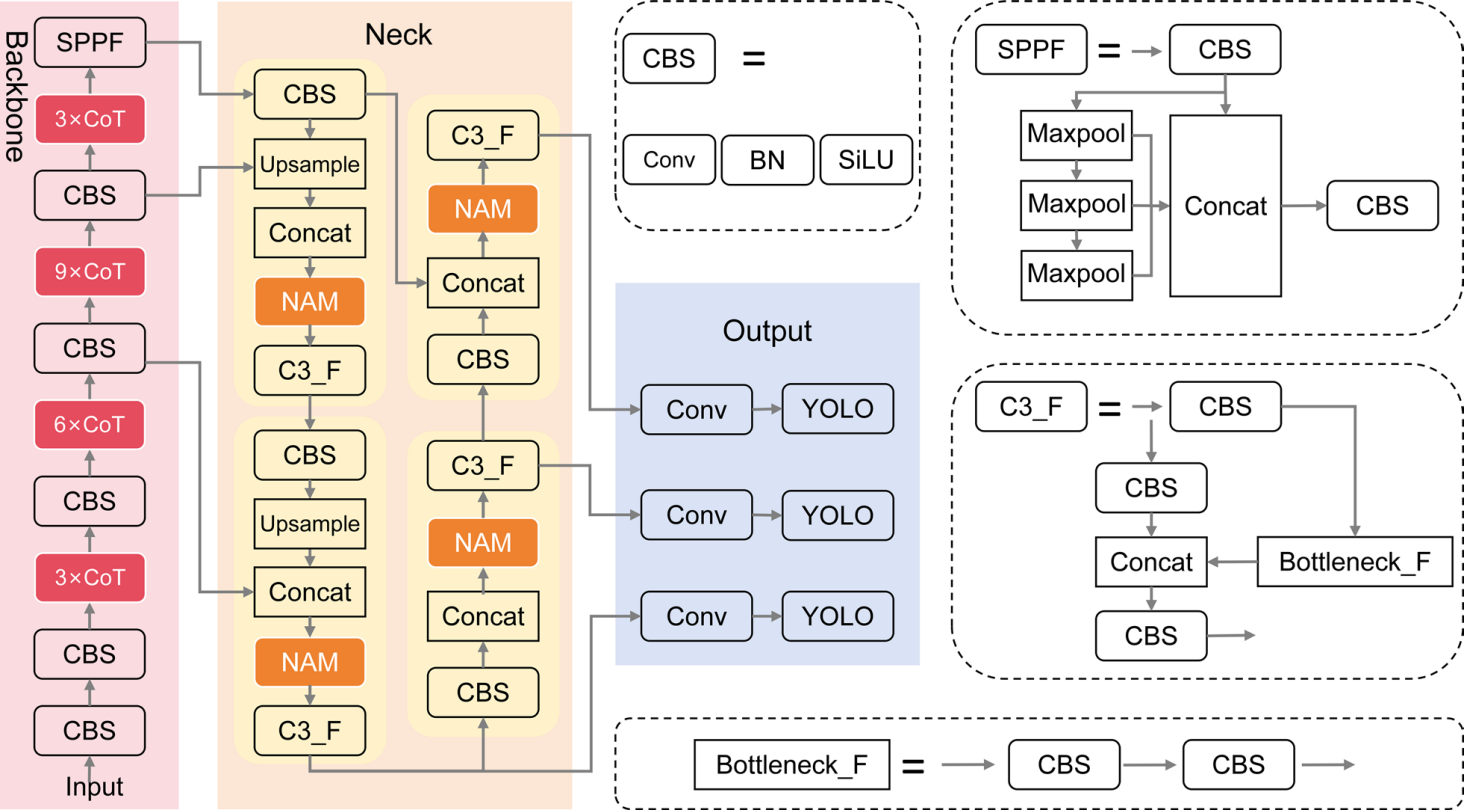
Architecture of the YOLO-GA detection model

In this study, we propose a globally-aware attention-enhanced YOLO detection model, named YOLO-GA, for the automated detection of *Eimeria* oocysts in microscopic images. As illustrated in Fig. 3, the model is built upon the YOLOv5 architecture [22], which is able to extract pixel-level contextual semantic information. YOLOv5 consists of three main components: a backbone, a neck and a detection head. The backbone employs a Cross Stage Partial (CSP) structure to improve training efficiency and reduce computational cost; the neck adopts a Path Aggregation Network (PANet) for multi-scale feature fusion; and the head generates final detection outputs. YOLOv5 was selected as the baseline framework because of its stable architecture, open-source availability and flexibility for integrating new modules. Compared to newer versions, such as YOLOv8 or YOLOv10, YOLOv5 offers a well-documented structure and community support, which facilitates modular experimentation. Moreover, the proposed YOLO-GA model has been benchmarked against YOLOv10 (Table 1), which demonstrated its superior detection performance. In this study, the YOLOv5s variant (small version) was used as the base model due to its balance of detection accuracy and computational efficiency, which is suitable for deployment in real-world veterinary diagnostic applications.

Based on YOLOv5, this study integrates two key modules: the CoT module [23] for global context awareness and the NAM [24] for refined attention distribution based on normalization mechanisms. The CoT module and the NAM module were adopted directly from the Contextual Transformer Networks for Visual Recognition and Normalization-based Attention Module, respectively, and integrated into the YOLOv5 architecture. The CoT module is embedded into the backbone and enhances feature representation of small objects (such as oocysts) by fusing static convolutional context with dynamic attention, thereby establishing a bidirectional interaction between local details and global semantics. The NAM module is incorporated into the neck, where it applies both channel-wise and spatial normalization-based attention to optimize the allocation of attention during feature fusion, thereby strengthening the network’s focus on informative regions. Through the combined effect of these two modules, YOLO-GA effectively addresses the common challenges in microscopic image analysis, such as the difficulty in detecting small targets and interference from complex backgrounds. As a result, the model achieves improved detection performance of *Eimeria* oocysts under challenging visual conditions.

### CoT module structure

To enhance the perception capability of the target detection model for small objects in complex backgrounds, this paper introduces the CoT module into the backbone network of YOLOv5, replacing a portion of the original C3 modules. This replacement improves contextual modeling and semantic feature extraction. Unlike the traditional C3 module, the CoT block incorporates a dynamic attention mechanism alongside static local convolutional features, enabling adaptive learning of dependencies between different spatial positions. This enhancement strengthens the model’s ability to model fine-grained targets.

As illustrated in Additional file 5: Figure S5, the CoT module consists of four components: a linear projection layer, a static context-encoding module, a dynamic attention generation module and a context fusion module. First, the input feature map undergoes linear transformations to generate query (*Q*), key (*K*) and value (*V*) vectors for subsequent context-aware processing. Next, depthwise separable convolution is applied to the key vector to encode static semantic information from local spatial regions, thereby enhancing perception of critical spatial relationships. The contextually-enriched key is then concatenated with the original query and fed into a lightweight attention generator composed of two cascaded 1 × 1 convolutional layers. Finally, the dynamically generated attention weights are fused with the value vector, producing a feature map that integrates both static semantics and dynamic attention information.

Let the input feature map to the backbone network as $X \in {\mathbb{R}}^{H\times W\times C}$, where *H*, *W* and *C* represent height, width and channel dimensions, respectively. The CoT block first generates query, key and value tensors via three linear transformations:

1 $$Q = X{W}_{Q}, K = X{W}_{K}, V = X{W}_{V},$$

where ${W}_{Q},{W}_{K},{W}_{V} \in {\mathbb{R}}^{{\varvec{C}}\boldsymbol{ }\times \boldsymbol{ }{\varvec{d}}}$ are learnable projection matrices, and *d* is the embedding dimension.

Subsequently, to extract local context, a $k \times k$ depthwise separable convolution is applied to the key tensor:

2 $${K}_{c} = {\text{DWConv}}_{k \times k}(K),$$

where DWConv denotes depthwise separable convolution, efficiently modeling spatial feature interactions while maintaining computational efficiency.

The query Q and contextually enriched key ${K}_{c}$ are concatenated along the channel dimension. This concatenated feature map is then passed through two $1 \times 1$ convolutional layers and a sigmoid activation to generate attention weights:

3 $$F =\text{ Concat}(\text{Q},{\text{K}}_{\text{c}}), A =\upsigma ({\text{Conv}}_{1 \times 1}^{(2)})(\text{ReLU}({\text{Conv}}_{1 \times 1}^{(1)}(F))),$$

where $A \in {\mathbb{R}}^{H \times W \times D}$ represents the attention map, and $\upsigma$ is the sigmoid function for normalization.

The attention map *A* is element-wise multiplied with the value vector *V* to obtain dynamic contextual features:

4 $$\text{Y }=\text{ A }\odot \text{ V},$$

where ⊙ denotes the Hadamard product.

Finally, the dynamic features *Y* are fused with the static context ${K}_{c}$. A $1 \times 1$ convolution and batch normalization (BN) are applied, followed by a residual connection to stabilize gradient flow:

5 $$O =\text{ BN}({\text{Conv}}_{1 \times 1}(Y + {K}_{c})) + X.$$

The output $O \in {\mathbb{R}}^{H \times W \times C}$ retains the same dimensions as the input, ensuring seamless integration with subsequent modules.

### NAM module structure

Microscopic parasite eggs in fecal samples often exhibit low contrast and complex backgrounds, leading to missed detections. To address this, we introduce a lightweight NAM into the neck of the YOLO model (Additional file 6: Figure. S6). As shown in Additional file 7: Figure S7, NAM sequentially combines channel attention and spatial attention mechanisms [25], directing the model to focus on salient regions while suppressing irrelevant background fluctuations.

For channel attention, the scaling factors from BN are utilized to model channel-wise importance. Given an input feature map $F \in {\mathbb{R}}^{C \times H \times W}$, the channel attention weight ${M}_{c}$ is computed as:

6 $${\mu }_{c} = \frac{1}{HW}\sum_{h=1}^{H}\sum_{w=1}^{W}{F}_{c}(h,w), {\sigma }_{c} = \sqrt{\frac{1}{HW}\sum_{h=1}^{H}\sum_{w=1}^{W}{(F}_{c}(h,w)-{\mu }_{c}{)}^{2}}$$

7 $${M}_{c} = \sigma ({\gamma }_{c}\bullet {\sigma }_{c}+{\beta }_{c})$$

where ${\gamma }_{c}$ and ${\beta }_{c}$ are BN parameters, and $\upsigma (\bullet )$ is the sigmoid function.

For spatial attention, the variance across channels at each spatial position $(h,w)$ is computed to measure saliency:

8 $${\sigma }_{hw} = \sqrt{\frac{1}{C}\sum_{c=1}^{C}({F}_{c}(h,w)-{\mu }_{hw}{)}^{2}}, {\mu }_{hw} = \frac{1}{C}\sum_{c=1}^{C}{F}_{c}(h,w)$$

9 $${M}_{hw} = \sigma ({\gamma }_{hw}\bullet {\sigma }_{hw}+{\beta }_{hw})$$

The final output *F*″ is obtained by sequentially applying channel and spatial attention weights:

10 $$F^{\prime } = M_{c} \otimes F, \quad F^{\prime \prime } = M_{hw} \otimes F^{\prime }$$

where ⊗ denotes broadcasted element-wise multiplication. The NAM module enhances focus on critical regions, particularly improving small object detection.

The introduction of the NAM module enhances the model's ability to focus on salient regions during the neck stage, which is particularly advantageous for small object detection tasks. Experimental results demonstrate that the model with NAM exhibits stronger target localization capability and improved robustness in the recognition of parasite oocysts.

### Evaluation metrics

To comprehensively evaluate the performance of the YOLOv5 model in detecting *Eimeria* oocysts, the following evaluation metrics were employed:

- *Precision*: Precision measures the proportion of true positive samples among all samples predicted as positive.
  11 $$\text{Precision}=\frac{\text{TP}}{\text{TP}+\text{FP}}$$.
  TP (true positives) refers to correctly predicted positive cases, FP (false positives) refers to incorrectly predicted positive cases, TN (true negatives) refers to correctly predicted negative cases and FN (false negatives) refers to incorrectly predicted negative cases.
- *Recall*: Recall measures the proportion of actual positive samples that are correctly identified by the model.
  12 $$\text{Recall}=\frac{\text{TP}}{\text{TP}+\text{FN}}$$.
  Recall reflects the model’s ability to identify positive cases. A higher recall indicates a lower miss rate and better detection completeness.
- *Average Precision (mAP)*: mAP is a commonly used metric in multi-class object detection tasks to evaluate the overall detection performance across all classes.
  13 $$\text{mAP}=\frac{1}{N}\sum_{i=1}^{N}{\text{AP}}_{i}$$.
  In this study, only a single class (*k* = 1) is involved, so the AP is equivalent to mAP. We report mAP_50, which represents the detection accuracy at the standard Intersection over Union (IoU) threshold of 0.5 [26]. This metric provides an intuitive and representative evaluation of model performance and allows for effective comparison with other methods.
- *F1-score*: In addition, this study also employed the F1-score [27] as an evaluation metric to assess the overlap quality of predicted bounding boxes. The F1-score is the harmonic mean of precision and recall, combining both metrics into a single measure that balances their trade-off:
  14 $$\text{F}1\_\text{score}=2\times \frac{\text{Precision}\times \text{Recall}}{\text{Precision}+\text{Recall}}$$.
  The F1-score provides a comprehensive reflection of the model’s performance, especially in scenarios where both precision and recall are important.

### Experimental setup

This study conducted model training and inference on a Dell Precision 7920 Tower workstation (Dell Technologies, Round Rock, TX, USA) running the Ubuntu operating system. The system was equipped with two NVIDIA GeForce RTX 3090 GPUs (total VRAM: 48 GB), utilizing CUDA 12.4 and NVIDIA driver version 550.67 for GPU acceleration. The model was developed under the PyTorch 2.0.1 framework, leveraging GPU-accelerated parallel computing to enhance training efficiency and detection performance. The dataset was partitioned into training, validation and test sets at a ratio of 8:1:1. During training, the Adam optimizer was employed with an initial learning rate of 0.001, a batch size of 16 and a total of 100 epochs.

## Results and discussion

### Experimental result

**Fig. 4:**
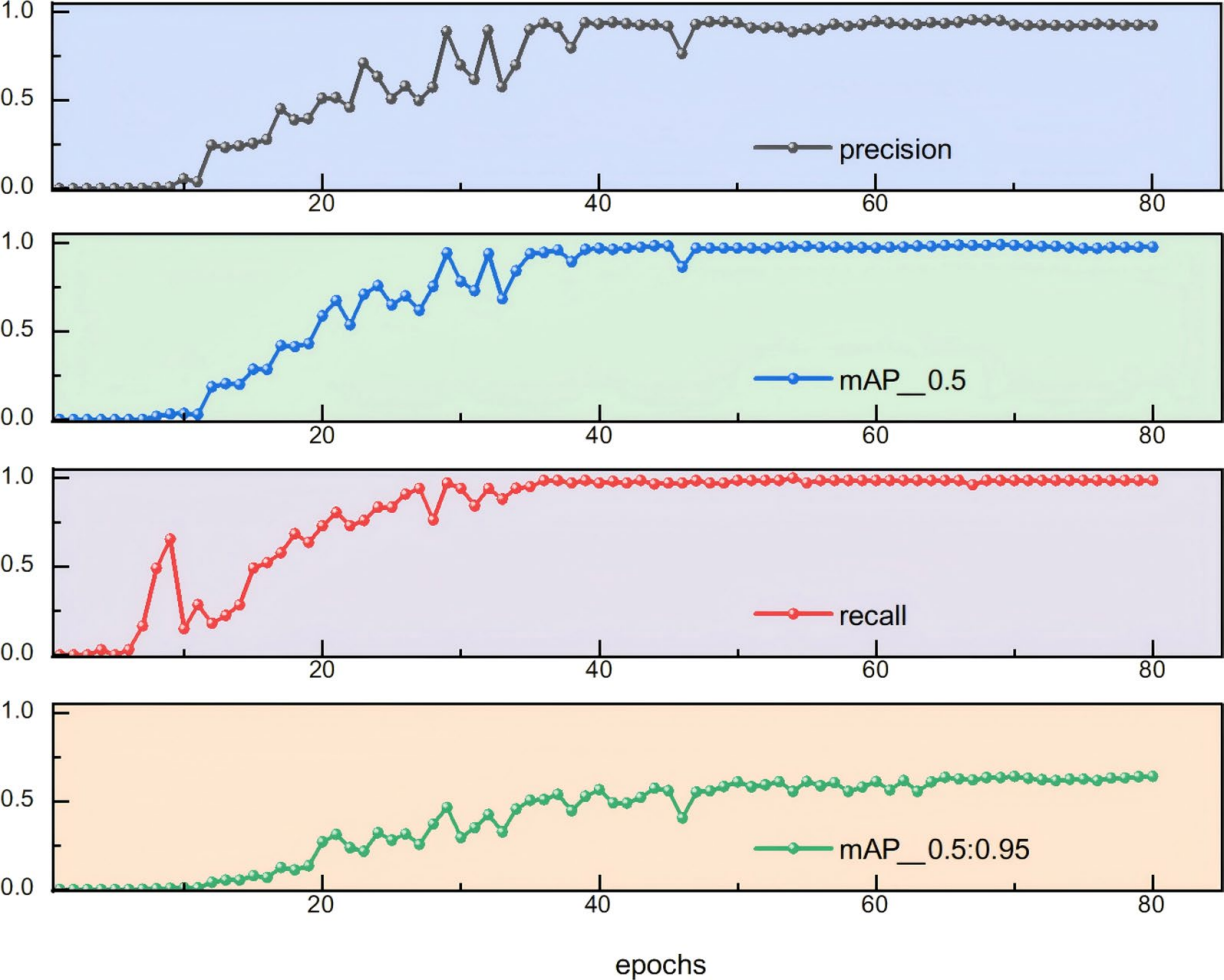
Model performance curves. From top to bottom, the plots illustrate the changes in precision, mAP@0.5, recall and mAP@0.5:0.95 over training epochs, providing a comprehensive view of the model's performance evolution during training

The performance curves of the proposed detection model in the *Eimeria* oocyst detection experiment are shown in Fig. 4. These curves illustrate the trends of several key performance metrics during the model validation process. Notably, the validation dataset was not used for training and serves purely to reflect the model’s performance throughout training.

As shown in Fig. 4, with the increase in training iterations, the values of precision, recall and mAP exhibit a steady upward trend. During the initial training phase (epochs 0–40), these metrics show noticeable fluctuations, yet the overall trajectory remains positive. Between epochs 30 and 50, the fluctuations begin to stabilize, with the curves leveling off progressively. After approximately 50 epochs, all curves tend toward convergence, appearing nearly linear. This behavior can be attributed to the iterative refinement of model weights during training, which gradually reduces the discrepancy between predictions and ground truth. Since parameter updates are performed on a batch-wise basis, a single batch may not yield immediate performance improvements. Instead, the model incrementally learns representative features across multiple batches. This dynamic learning process often introduces temporary fluctuations in training loss, but the overall trend is a consistent decline. As training progresses, the model approaches its optimal state, with diminished adjustments to parameters and a correspondingly smaller variation in performance metrics. Specifically, the mAP curve transitions from a phase of oscillatory growth to one of minor fluctuations, ultimately stabilizing at a value of 0.989. In summary, the model achieved strong performance on this single-class dataset. The peak values of precision, recall, mAP@0.5 and mAP@0.5:0.95 reached 0.952, 0.985, 0.989 and 63.3, respectively, indicating high annotation quality and well-tuned training hyperparameters.

### Methods comparison

To comprehensively evaluate the performance of the proposed model in the detection of *Eimeria* oocysts, several mainstream object detection models were selected as comparative baselines, including YOLOv3 [28], YOLOv5, YOLOv6 [29], YOLOv7 [30], YOLOv8 [31], YOLOv10 [32] and DETR [33]. These models represent various stages in the development of the YOLO architecture and include a recent transformer-based detector, thereby providing a broad and meaningful benchmark for comparison. All models were evaluated under identical experimental settings using the same dataset. The experimental results are summarized in Table 1.

**Table 1:** Performance comparison of YOLO-GA and state-of-the-art detection models on the *Eimeria* oocyst dataset

Model	Parameters (M)	GFLOPs	Precision (%)	Recall (%)	F1-score (%)	mAP@0.5 (%)	mAP@0.5:0.95 (%)
YOLOv3	61.50	154.5	91.1 ± 0.3	97.0 ± 0.2	93.9 ± 0.2	96.6 ± 0.4	59.9 ± 0.5
YOLOv5	7.01	15.8	94.3 ± 0.2	97.0 ± 0.1	95.6 ± 0.2	98.1 ± 0.2	53.3 ± 0.4
YOLOv6	20.11	24.9	91.3 ± 0.4	96.7 ± 0.3	93.9 ± 0.3	93.8 ± 0.6	60.0 ± 0.6
YOLOv7	36.48	103.2	94.1 ± 0.2	95.5 ± 0.3	94.8 ± 0.2	97.2 ± 0.3	65.6 ± 0.5
YOLOv8	11.14	28.6	92.9 ± 0.3	*98.5 ± 0.2*	95.6 ± 0.3	97.5 ± 0.2	*67.3 ± 0.3*
YOLOv10	8.04	24.4	90.8 ± 0.5	91.4 ± 0.4	91.1 ± 0.4	94.8 ± 0.5	57.7 ± 0.6
DETR	36.74	57.11	91.4 ± 0.4	55.9 ± 0.6	69.1 ± 0.5	95.2 ± 0.3	43.8 ± 0.4
YOLO-GA	*6.99*	*15.7*	*95.2 ± 0.3*	*98.5 ± 0.2*	*96.8 ± 0.2*	*98.9 ± 0.1*	63.3 ± 0.3

Results are reported as mean ± standard deviation over three independent runs

*GFLOPs* Gigaflops (1 billion floating-point operations s^−1^),* mAP* mean average precision. Italics are used to indicate the results of the method we proposed.

In Table 1, the reported values for precision, recall, F1-score and mAP are presented as means ± standard deviations (SD) across three independent runs, reflecting the model's performance stability and reliability. Within the YOLO series, all models achieved recall rates, precision and mAP@0.5 values above 90%, indicating that each model was capable of accurately detecting *Eimeria* oocysts. However, significant performance differences across models remain evident. In terms of precision, the proposed YOLO-GA model outperformed all others, reaching 95.2%, demonstrating its strong capability in reducing false positives. YOLOv5 and YOLOv7 also exhibited high precision (94.3% and 94.1%, respectively), while YOLOv3, YOLOv6, YOLOv8 and YOLOv10 showed relatively consistent performance around 91%, albeit slightly lower than the top performers. Regarding recall, both YOLOv8 and YOLO-GA achieved the highest value of 98.5%, reflecting their outstanding ability to detect nearly all target instances. YOLOv3, YOLOv5 and YOLOv6 also performed well, with recall values ranging from 96.7% to 97.0%. The F1-score, which balances precision and recall, provides a more comprehensive indicator of detection quality. YOLO-GA again led with an F1-score of 96.8%, followed closely by YOLOv5 and YOLOv8, each at 95.6%, indicating that YOLO-GA strikes the best trade-off between detection accuracy and completeness. In terms of mAP@0.5, YOLO-GA achieved the highest score at 98.9%, followed by YOLOv5 (98.1%) and YOLOv8 (97.5%), confirming their strong target recognition ability under lenient localization thresholds. However, when considering mAP@0.5:0.95, which averages performance across a range of Intersection over Union (IoU) thresholds and emphasizes localization precision, YOLOv8 achieved the best score at 67.3%. This suggests its superior bounding box regression accuracy. By comparison, YOLOv5 reached only 53.3%; despite high mAP@0.5 performance, its detection precision drops under stricter IoU conditions. YOLO-GA’s mAP@0.5:0.95 was 63.3%, ranking it in the upper-middle tier. This demonstrates that while prioritizing high F1-score and mAP@0.5, YOLO-GA also maintains a respectable level of localization accuracy.

DETR, being a transformer-based model, showed comparable precision and mAP@0.5 values to the YOLO models, but its recall (55.9%) and mAP@0.5:0.95 (43.8%) were lower. This highlights the challenges of applying transformer-based models to this task, especially when localization precision is critical. Parameters (M) and GFLOPs are important metrics for evaluating model computational efficiency and resource consumption. YOLO-GA, with the least number of parameters and computations, improves inference efficiency while maintaining high precision. On the other hand, DETR, as a transformer-based model, performs well in mAP@0.5, but due to its higher computational demands, it may not be as suitable for resource-constrained environments compared to the YOLO series. This further highlights the well-balanced overall performance of YOLO-GA.

**Fig. 5:**
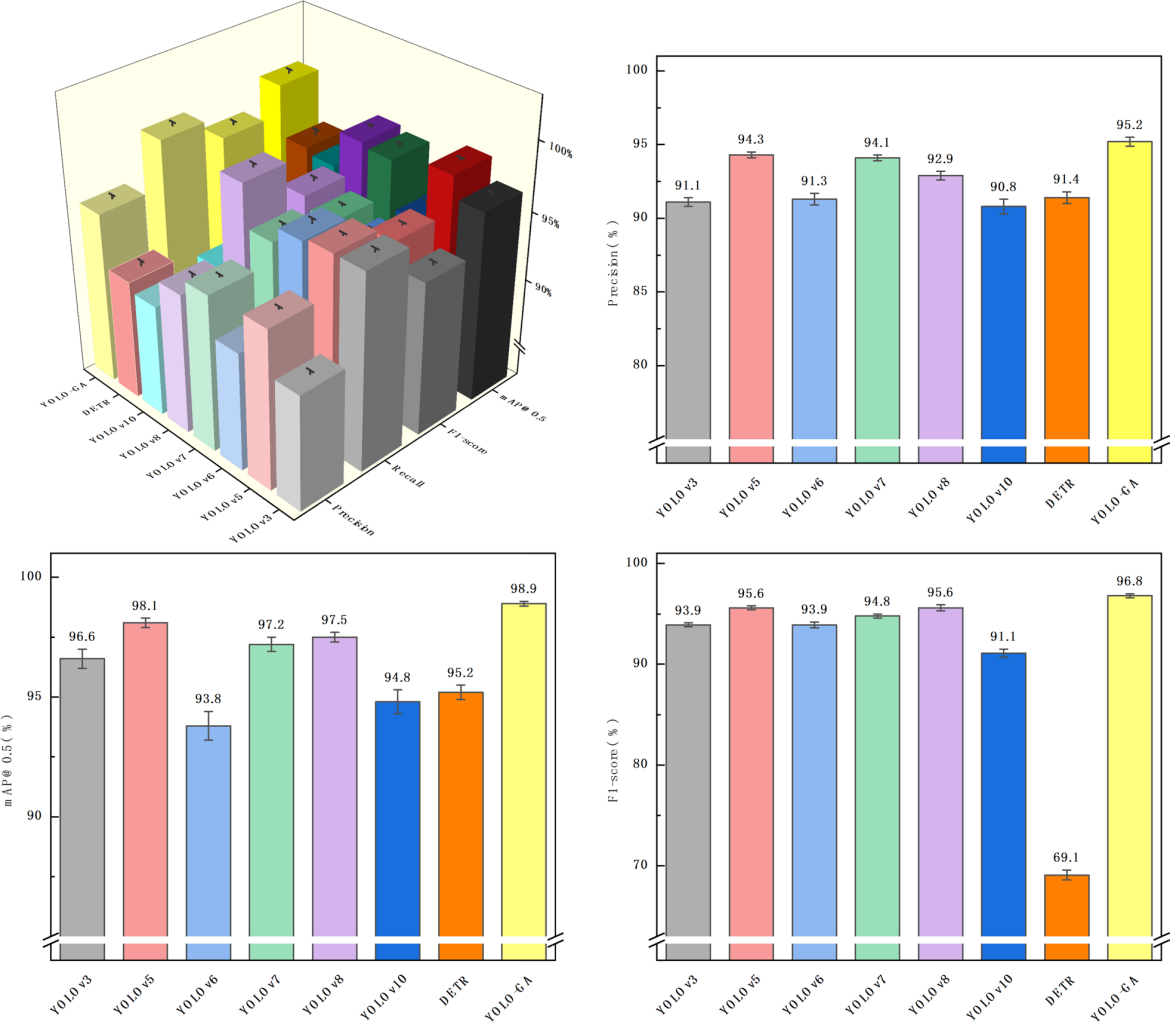
Quantitative performance comparison of different object detection models: precision, recall, mAP@0.5 and F1-score

Additionally, Fig. 5 provides a visual comparison of the eight models across various evaluation metrics. The YOLO-GA model consistently outperformed its counterparts, especially under higher IoU thresholds, where its mAP values were significantly superior, indicating enhanced capability in precise object localization. Furthermore, YOLO-GA maintained high mAP across a range of IoU thresholds, demonstrating balanced detection across targets of different sizes. It also outperformed other models in terms of precision, recall, and F1-score, further validating its robust and effective detection capabilities. In conclusion, on our specific dataset, the YOLO-GA model exhibited an advantage in key detection metrics, suggesting improved reliability and precision for this task under the tested conditions. Other models, while performing well, showed varying degrees of inferiority compared to YOLO-GA across most key metrics.

### Visualization analysis

**Fig. 6:**
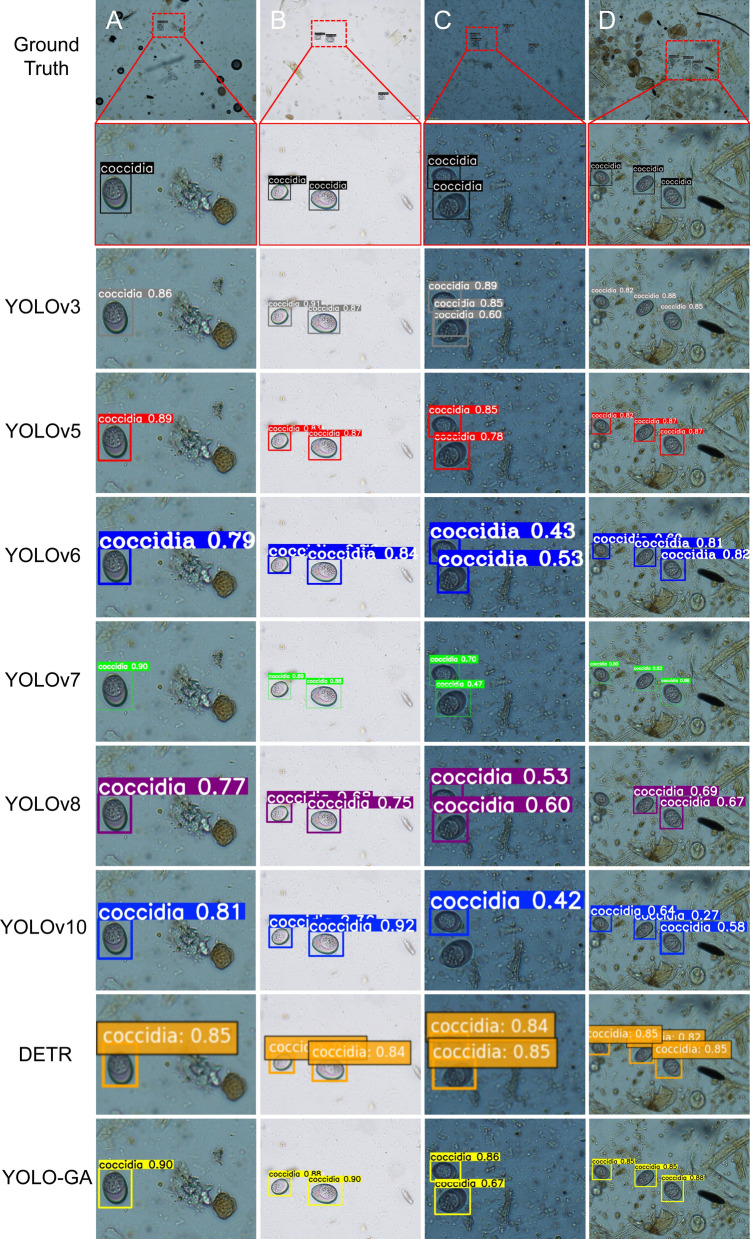
Comparative visualization of *Eimeria* oocyst detection under varying conditions: **a** Single target in a complex background, **b** multi-target in a simple background, **c** occluded targets, **d** multi-target in a cluttered environment. Confidence scores are normalized to the [0, 1] scale

To intuitively demonstrate the detection performance of the model, Fig. 6 presents the detection results of *Eimeria* oocysts in ovine fecal samples under a microscope using eight different object detection methods. The first row of Fig. 6 displays the distribution of parasites under four different image conditions, with varying sizes and background complexities. Figure 6a shows the detection of a single oocyst in a complex background with visual interference. Among the eight models, YOLOv3, YOLOv6 and YOLOv8 performed relatively poorly. The remaining five models yielded comparable results, with confidence scores exceeding 0.80 for all. Figure 6b illustrates the detection of multiple oocysts in a simple background. In this scenario, all models, except YOLOv8, demonstrated strong robustness, with seven models achieving confidence scores above 0.80. Only models YOLOv5, YOLOv6 and DETR had a few predictions with scores below 0.85. The proposed YOLO-GA model performed on par with YOLOv10, both achieving the highest confidence scores in this case. Figure 6c presents a more challenging scenario where targets are partially overlapping or closely spaced. In this case, the overall detection performance was suboptimal across all models. For YOLOv6, YOLOv7 and YOLOv8, the highest confidence score for the two targets was only 0.70, with the lowest model having a score of 0.43; most predictions hovered around 0.50. YOLOv10's confidence score for the targets was only 0.42, and even missed some detections. Although YOLOv3 showed higher confidence, it also produced many false positives. Only YOLO-GA and YOLOv5 accurately detected the oocysts with acceptable confidence levels, indicating better reliability when handling occlusion or crowding. Figure 6d shows the detection of multiple oocysts in a complex background. The presence of multiple targets and a complex background increases the detection difficulty. Interestingly, all models, except YOLOv8, successfully identified the oocysts, with YOLO-GA, YOLOv3, YOLOv5 and DETR showing similar confidence scores around 0.85. Across both complex backgrounds and multi-target scenarios, YOLO-GA consistently demonstrated stable performance, highlighting its strong robustness. This model remained among the top-performing models in terms of confidence scores across all test images. This consistency indicates that YOLO-GA can reliably detect targets under varying levels of interference, reflecting its strong adaptability and generalization ability.

### Visualization of model attention and interpretability

**Fig. 7:**
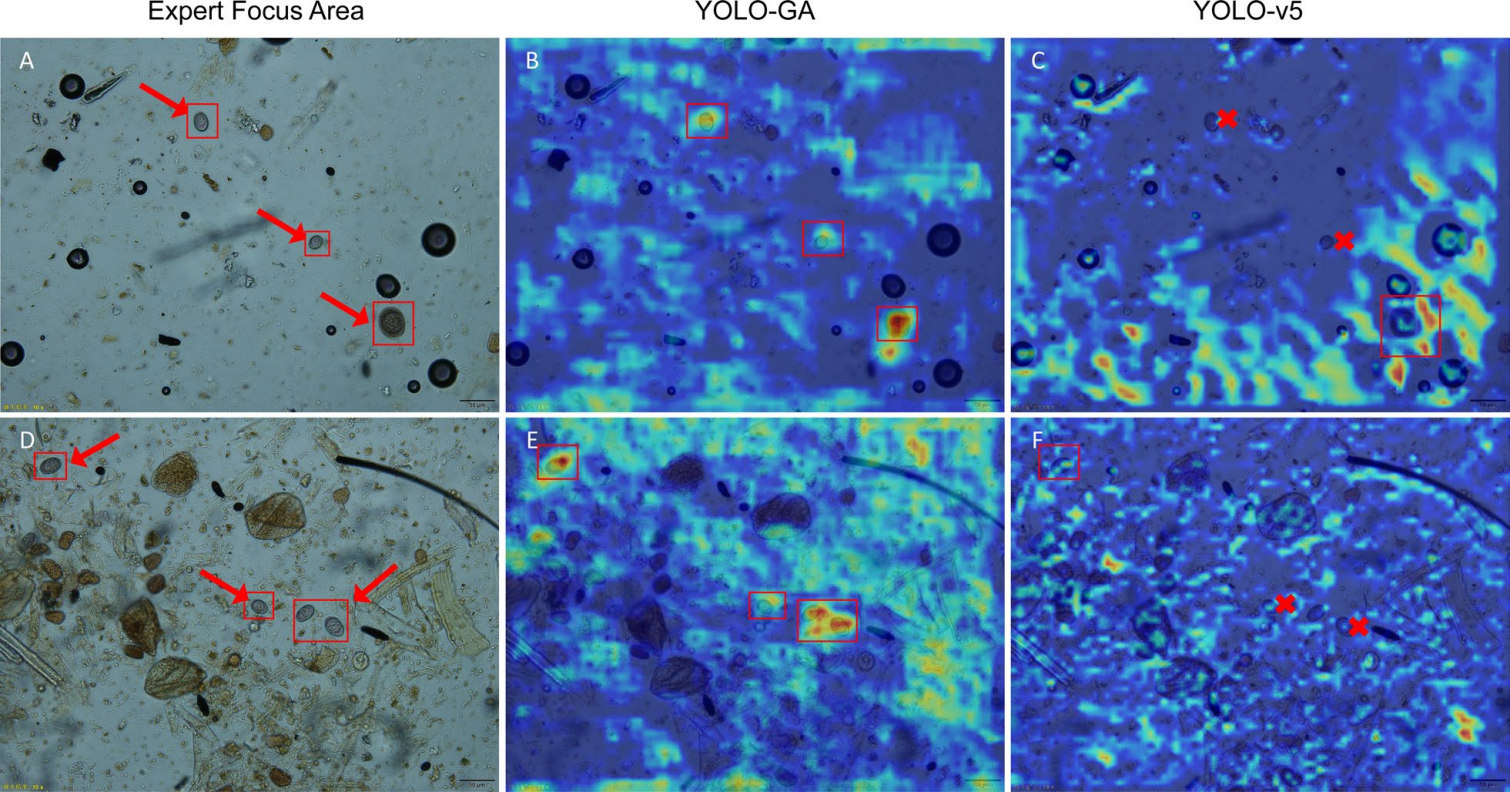
Comparison of attention regions between experts and models. In panels **a**, **d** Manual annotations from the two veterinary experts, with the arrows indicating the regions manually annotated by parasitology experts. **b**, **e** Heatmaps produced by model YOLO-GA, with the red boxes highlighting the high-response areas identified by the YOLO-GA model. **c**, **f** Heatmaps produced by model YOLOv5, with the crosses denoting regions that YOLOv5 failed to attend to but which were considered diagnostically relevant by experts

To further explore the interpretability of the proposed YOLO-GA model, we visualized its attention regions using class activation maps (CAM) [34]. The CAM results were compared with expert-annotated regions to assess the model’s ability to focus on biologically relevant areas. Two veterinary experts with extensive experience in parasitic disease diagnosis were invited to manually annotate the *Eimeria* oocysts in the test images. Their annotations included the spatial locations of the oocysts and morphologically significant regions, which served as the “ground-truth” expert attention areas. Subsequently, the CAM-based heatmaps generated by the YOLO-GA model on test samples were processed and compared with the expert-labeled areas to assess spatial overlap and consistency in salient region focus. Figure 7a, d shows the manual annotations from the two veterinary experts, where red arrows highlight the annotated oocyst regions, including both their positions and morphological features. Figure 7b, e presents the heatmaps produced by YOLO-GA, with red bounding boxes indicating the model's focus areas. Figure 7c, f presents the heatmaps produced by YOLOv5. These heatmaps visualize the degree of model attention across different regions in the images using CAM.

As illustrated in Fig. 7, the high-response regions in the YOLO-GA-generated heatmaps that significantly overlap with the expert-labeled regions, indicating that the model accurately localizes the oocysts. Compared to YOLOv5, the YOLO-GA model exhibits a closer alignment between its attention regions and the morphologically significant areas identified by experts, suggesting that YOLO-GA possesses a stronger ability to capture key oocyst features. This superior focus can be largely attributed to the integration of the CoT and NAM modules. The CoT module enhances the model’s capacity to capture both local and global contextual information by combining static convolutional context with dynamic attention mechanisms. This allows YOLO-GA to better detect subtle features in microscopic images. Meanwhile, the NAM refines the attention distribution across both channel and spatial dimensions, enabling the model to more accurately focus on biologically relevant features such as *Eimeria* oocysts.

### 3D Visualization of model attention

Additional file 8: Figure S8 presents a 3D class activation heatmap generated by the YOLO-GA model, superimposed on the original microscopic images to detect *Eimeria* oocysts [35, 36]. On the left, the 3D plot illustrates the Grad-CAM activation map, where the microscopic image is projected into 3D space based on activation intensity. Both the color and elevation of the plot reflect the strength of the model’s attention to specific regions. In the upper right, veterinary experts have manually annotated the oocyst positions on the original images, while the lower right shows the corresponding 2D activation heatmap produced by the model.

A comparison of the results clearly demonstrates that the YOLO-GA model’s main regions of interest—highlighted as red peaks—closely coincide with the expert-annotated positions. This indicates the model’s ability to autonomously attend to diagnostically relevant areas even without explicit guidance. Furthermore, the model exhibits minimal activation in non-target regions, represented by flat blue-green areas, effectively suppressing false positives arising from background noise or irrelevant tissue components. This sparse activation pattern is consistent with the diagnostic behavior of clinical experts, who typically focus solely on suspicious parasitic structures.

Such alignment not only showcases the YOLO-GA model’s robust capacity for detecting small targets in microscopic imagery but also indirectly verifies the synergistic effectiveness of the CoT module and the NAM in feature extraction and spatial focus. The CoT module enables the model to comprehend relationships between local and global contexts, allowing it to isolate relevant information from densely cluttered backgrounds, while the NAM module enhances spatial selectivity, mimicking the expert-like process of progressively filtering salient parasitic features.

In conclusion, these visualization results highlight the interpretability and biological consistency of the YOLO-GA model, supporting its potential for use in intelligent assisted diagnosis in veterinary applications.

## Conclusions

This study proposed a deep learning-based object detection model, YOLO-GA, for the identification of *Eimeria* oocysts in microscopic images. Built upon the YOLOv5 framework, the model integrates CoT and NAM modules to enhance feature representation. Trained and validated on a dataset of 2000 images, YOLO-GA demonstrated strong performance, outperforming several state-of-the-art detectors on this specific task. These findings suggest that the model shows promise as an efficient tool for the automated detection of* Eimeria *oocysts. However, it is important to note that this study serves as a proof-of-concept; further validation is necessary to assess its efficacy as a practical tool in real-world veterinary applications, where sample variability and operating conditions can be significantly more complex.

Despite the promising results, this work has several limitations that must be acknowledged to accurately contextualize the findings. First, the dataset, while well-annotated, has limitations in terms of its diversity and complexity. The images primarily contain a low-to-moderate number of oocysts, with few examples of densely clustered or severely overlapping targets. This may limit the model's robustness and generalization capability. Second, the performance gap between mAP@0.5 and mAP@0.5:0.95 warrants careful interpretation. The high mAP@0.5 indicates excellent performance at a lenient localization threshold, which is suitable for detection and counting, suggesting that the model is highly effective at finding oocysts but may be less accurate at delineating their exact boundaries. Third, the model's complexity was not reduced. The integration of the CoT and NAM modules enhanced performance but increased parameter count and computational load compared to the base YOLOv5s. Future work should focus on model compression and pruning. Finally, and most critically, the generalizability of the model remains unproven. The study was conducted on images acquired from a single source using a specific microscope and protocol. This limits the immediate practical applicability and underscores the need for external validation.

In future research, we plan to expand the dataset to include a wider range of challenging imaging conditions, explore model compression and lightweight optimization techniques to improve deployment efficiency and conduct cross-domain validation using diverse data sources to further enhance model robustness.

## Supplementary Information

**Addtional file 1: Figure S1.** Four augmented samples generated from a single original image using random data augmentation techniques, including rotation, scaling , horizontal and vertical flipping, brightness and contrast adjustment , color saturation disturbance and Gaussian noise. These augmentations aim to improve the robustness of the detection model under diverse imaging conditions.

**Additional file 2: Figure S2.** Dataset. Dataset overview.

**Additional file 3: Figure S3.** Annotation statistics. (a) Sample quantity and class distribution in the training set; (b) size and count distribution of bounding boxes; (c) spatial distribution of bounding box centers within the entire image; (d) aspect ratio distribution of targets relative to the image dimensions.

**Additional file 4: Figure S4.** Correlation matrix of annotation features. This correlogram illustrates the pairwise relationships between key bounding box attributes such as center coordinates, width and height, revealing the statistical dependencies in object shape and spatial distribution.

**Additional file 5: Figure S5.** Architecture of CoT.

**Additional file 6: Figure S6.** Architecture of NAM.

**Additional file 7: Figure S7.** Structures of the channel attention and spatial attention modules.

**Additional file 8: Figure S8.** 3D visualization of model attention using Grad-CAM, overlaid on the original microscopic image. The left panel shows a 3D activation map; the upper right shows expert annotations; the lower right shows a corresponding 2D activation heatmap.

## Data Availability

All the data generated or analyzed during this study are included in this published article.
