# Supplementary figures and images for: Precise detection of Eimeria oocysts in sheep: a deep learning model based on microscopic images
Source: Parasit Vectors. 2025 Nov 12;18:459. doi: 10.1186/s13071-025-07092-4 (PMC12613407; doi:10.1186/s13071-025-07092-4)

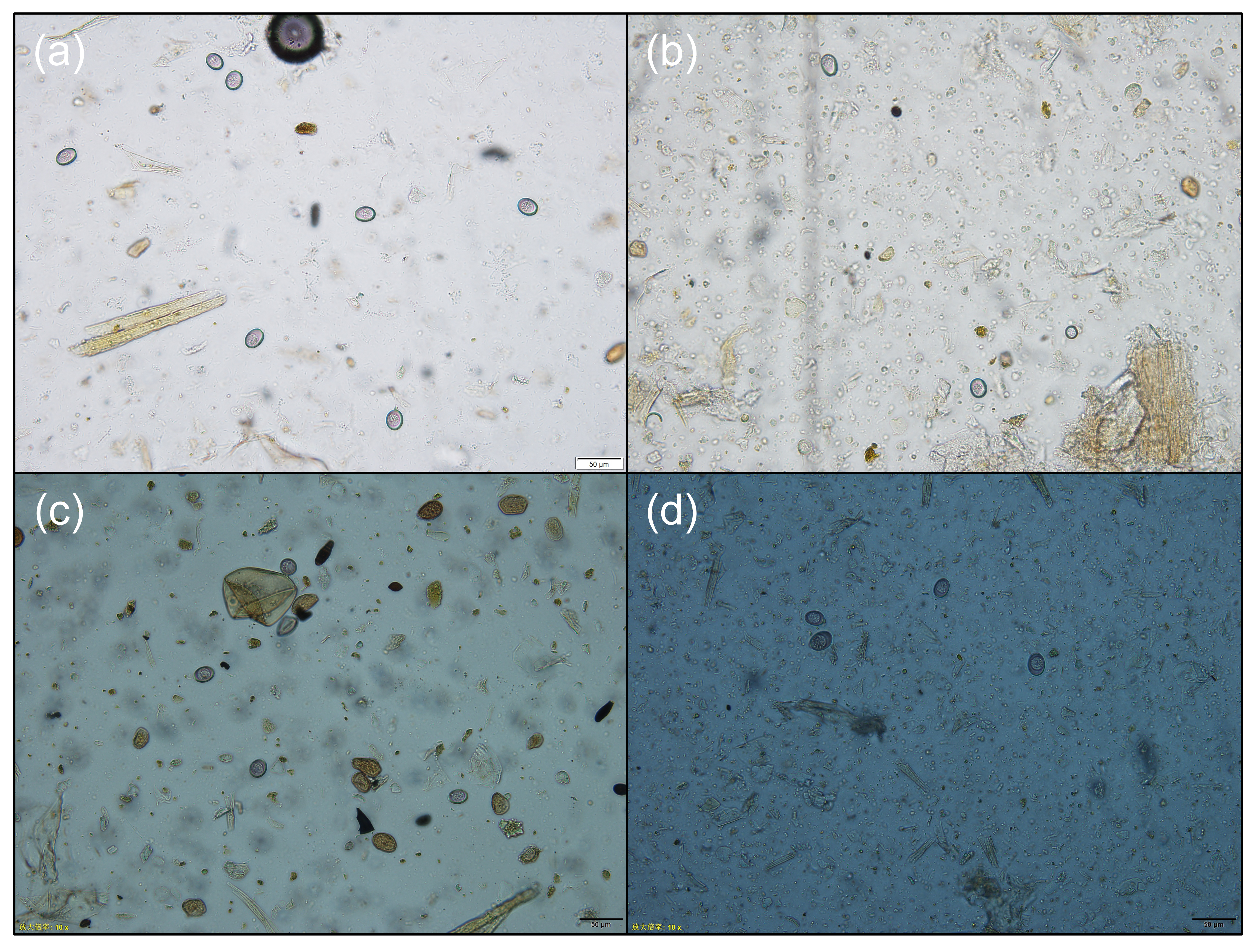

Supplement: Supplementary file 2 — Additional file 2: Figure S2. Dataset. Dataset overview. [file 13071_2025_7092_MOESM2_ESM.tif]

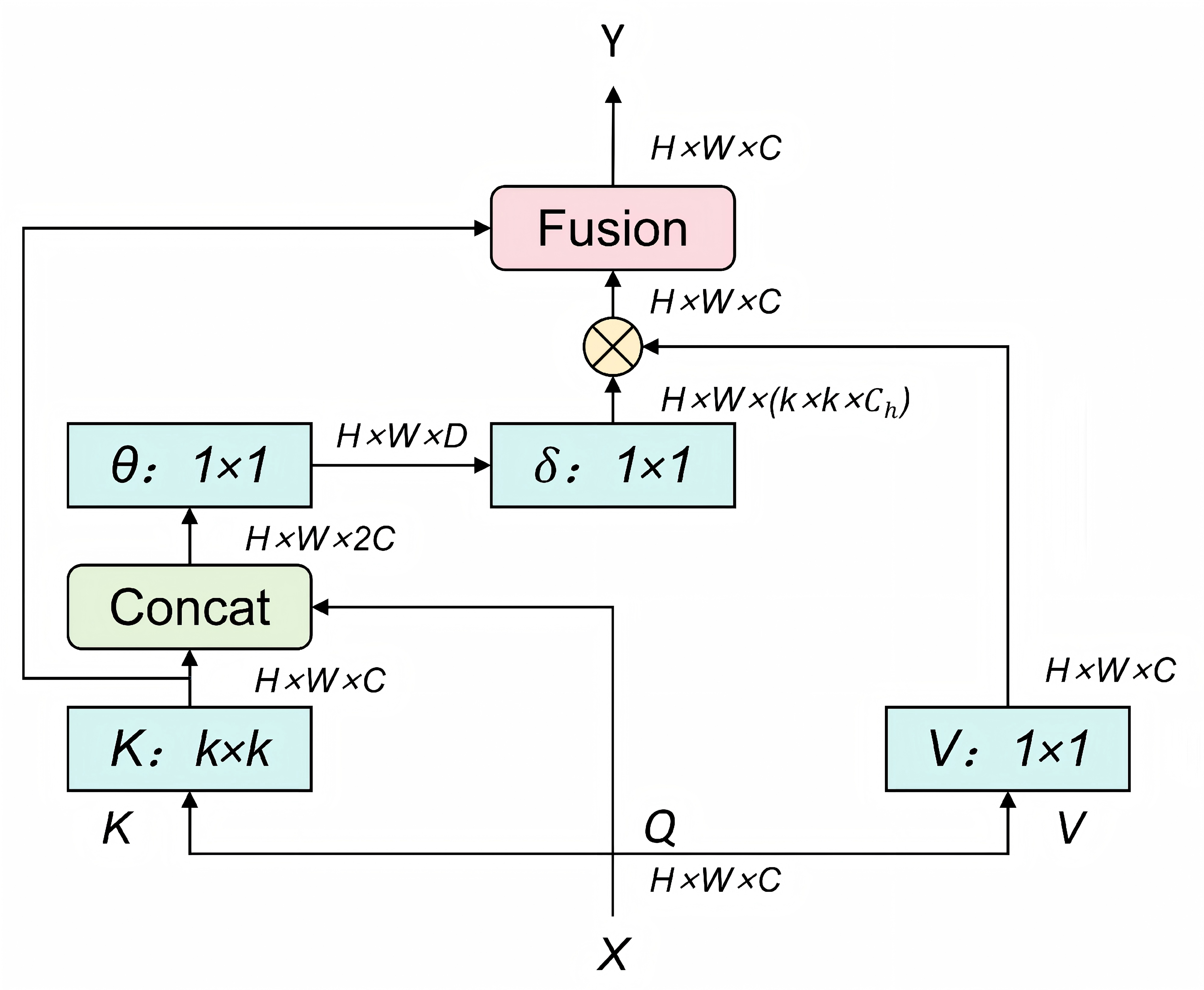

Supplement: Supplementary file 5 — Additional file 5: Figure S5. Architecture of CoT. [file 13071_2025_7092_MOESM5_ESM.tif]

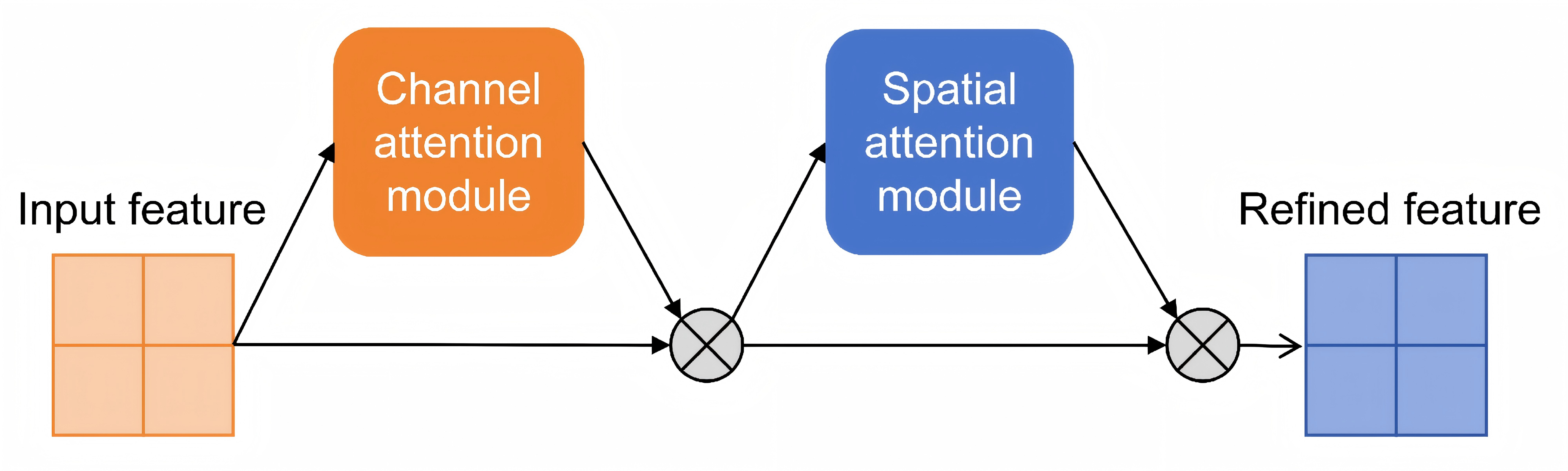

Supplement: Supplementary file 6 — Additional file 6: Figure S6. Architecture of NAM. [file 13071_2025_7092_MOESM6_ESM.tif]
